# Short-term outcome of percutaneous coronary intervention with directional coronary atherectomy followed by drug-coated balloon: a preliminary report

**DOI:** 10.1007/s12928-018-0537-6

**Published:** 2018-07-10

**Authors:** Akihiko Sato, Mikihiro Kijima, Shohei Ichimura, Daiki Yaegashi, Fumiya Anzai, Takeshi Shimizu, Yuko Matsui, Hironori Kaneko, Keiji Sakamoto, Yoshitane Seino, Yukio Maruyama, Yasuchika Takeishi

**Affiliations:** 1 Department of Cardiology and Vascular Medicine, Hoshi General Hospital, 159-1 Mukaigawara, Koriyama, Fukushima 963-8521 Japan; 2 Department of Cardiovascular Medicine, Fukushima Medical University, Fukushima, Japan

**Keywords:** Directional coronary atherectomy, Drug-coated balloon, Percutaneous coronary intervention

## Abstract

Directional coronary atherectomy (DCA) is a unique technique used in percutaneous coronary intervention (PCI) which involves the removal of plaque from the coronary artery. Treatment with a drug-coated balloon (DCB) appears to be effective, especially when a predilatation of the lesion is performed appropriately. We hypothesize that the combination therapy of DCA with DCB is an effective strategy in PCI. PCI with DCA followed by DCB was performed for 23 patients from December 2014 to April 2017. All DCA procedures were performed under the guidance of intravascular ultrasound (IVUS) findings and all procedures were successfully performed without incurring major complications such as a coronary perforation. Plaque area (PA) was reduced from 77.3 ± 10.4% at baseline to 50.9 ± 9.2% after DCA and luminal cross-sectional area (CSA) after PCI was enlarged from 3.6 ± 1.8 to 9.3 ± 3.3 mm^2^. Follow-up coronary angiography (CAG) performed at 6–10 months showed no cases having incurred restenosis. Plaque area at follow-up CAG was 52.0 ± 8.5% and luminal CSA was 9.5 ± 2.1 mm^2^. There were no cases undergoing target vessel revascularization (TVR) and target lesion revascularization (TLR) during the follow-up periods. PCI with DCA followed by DCB might be an effective strategy for de novo lesions.

## Introduction

Percutaneous coronary intervention (PCI) has been evolving with the emergence of new therapeutic devices since the first PCI was performed by Andrea Gruentzig in 1977, resulting in a continuous improvement in the outcome of PCI procedures for coronary heart disease. Directional coronary atherectomy (DCA), which is a unique technique that involves the removal of plaque from the coronary artery, was first performed in 1990 in the USA with the intent of reducing the abrupt closure and late restenosis rates after plain old balloon angioplasty (POBA). However, the first large randomized clinical trial (CAVEAT) conducted in 1991 failed to demonstrate superiority on the rate of late restenosis in the DCA group compared with the POBA group [1]. Although several clinical trials in the 1990s demonstrated optimal DCA supported by angiographical and intravascular ultrasound (IVUS) findings were superior compared with POBA on late restenosis rate [2–4], DCA was not generally accepted in western countries due to the complex nature of the procedure and given that coronary perforation was regarded as a critical complication of DCA. On the other hand, coronary intervention with bare metal stents commercialized in 1994 had demonstrated a dramatic reduction of acute abrupt closure and late restenosis compared with POBA [5], which DCA failed to demonstrate. Finally, 7 years after the commercialization of drug-eluting stent (DES) which reduced the rate of restenosis to less than 10% with a simple procedure [6], the DCA device was removed from the market in 2008. However, DES has not resolved all problems related to PCI, especially in regard to challenging bifurcated lesions [7]. Moreover, it was reported in 2007 that DCA was useful for select patients with bifurcated lesions including left main trunk [8]. Accordingly, a new DCA device was developed by NIPRO Corporation (Osaka, Japan) and became available in Japan in December 2014. After revival of a new DCA catheter, DCA has been performed for suitable coronary lesions such as non-tortuous and non-severe calcified lesions in proximal to middle segment with relatively large vessel diameter and short length (< 20–30 mm), especially bifurcated lesions including the left main trunk (LMT), ostial left anterior descending coronary artery (LAD) and ostial left circumflex coronary artery (LCX).

The drug-coated balloon (DCB) is a new device of coronary intervention and the usefulness of DCB in in-stent restenosis, bifurcated lesions and small vessel diseases is well established by several studies and many clinical experiences [9]. Recent studies have reported the clinical efficacy of stentless PCI with DCB for de novo lesions in DES era [10, 11], because PCI using DES leaves metal stents in a body and may cause stent-related adverse events such as stent thrombosis, neoatherosclerosis, metallic allergy and bleeding events associated with an antiplatelet therapy.

The removal of plaque with DCA might be compatible with DCB and PCI with DCA followed by DCB could be an effective option, which is not leaving metal stents in a body. The aim of this study was to investigate the effectiveness of PCI using DCA followed by DCB.

## Methods

This was a single-center, retrospective observational study conducted to evaluate the effectiveness of PCI using DCA followed by DCB (DCA–DCB). DCA–DCB was performed for 23 patients (23 lesions) with coronary heart disease and suitable lesion form for DCA from the time of revival of DCA in Japan (December, 2014) to April 2017, in our hospital. The DCA device used in this study was the ATHEROCUT catheter (Nipro Corporation, Osaka, Japan) and the indication of DCA–DCB was decided in accordance with angiographical findings, IVUS findings, the patient’s general condition (including renal function) and the judgment of experienced operators. Profiles of target lesion were non-tortuous and non-severe calcified lesions in proximal to middle segment with relatively large vessel diameter (> 2.5 mm) and short length (< 20–30 mm). IVUS-guided DCA via a trans-femoral approach with an 8F sheath was performed for all cases by experienced operators and the target residual plaque area after DCA was visual-estimated at less than 50% as previously reported [2]. The definition of deep cut in this study was the case with IVUS finding of the disruption of coronary arterial media caused by resection with DCA. Dilatation of the target lesion with DCB (SeQuent Please, Nipro Corporation, Osaka, Japan) was performed after DCA. The size of DCB was determined from the IVUS findings and inflation time of DCB was at least 30 s. Provisional stenting was planned in case of a coronary dissection occurring due to the DCA–DCB procedure, but was not performed in these 23 cases. Follow-up coronary angiography (CAG) and follow-up IVUS were performed at 6–10 months after PCI. Patient’s characteristics, lesion characteristics, procedural demographics including acute complications related to the PCI procedure, quantitative coronary angiography (QCA) and IVUS (QCU) data, and major adverse cardiac events (MACE) defined as composite events including cardiovascular death, non-fatal myocardial infarction, hospitalization due to unstable angina, target lesion revascularization (TLR) and target vessel revascularization (TVR) were all investigated during follow-up periods. QCA analysis was performed using the QAngio XA 7.3 (Medis Inc., Leiden, The Netherlands). Lesion length, reference diameter, minimal lumen diameter and diameter stenosis were measured. QCA analysis was conducted at timing of PCI and follow-up CAG. IVUS was performed with a commercially available system (Opticross, Boston Scientific, MA, USA) and QCU data were analyzed using QIVUS 2.1 (Medis Inc., Leiden, The Netherlands). Total vessel cross-sectional area (CSA), luminal CSA, and plaque area was measured. Antiplatelet therapy (APT) for patients treated with DCA–DCB was performed in accordance with the judgment of attending physicians.

Continuous variables were expressed as the mean ± SD and variable categories were expressed as frequencies. The protocol of this study was approved by the ethics committee of Hoshi General Hospital.

## Results

DCA was performed for 40 patients (40 lesions) from December 2014, to April 2017.

Out of the 40 patients, 23 patients were treated with DCA–DCB and follow-up CAG was performed for 14 patients. Seventeen of 40 patients received the planned implantation of DES following DCA because of diffuse or long lesions which was unfavorable for DCA–DCB or history of implantation of DES at the distal site of DCA. In 17 cases with DES following DCA, there was no case that received provisional stenting due to DCA-related complications such as major coronary dissections. The mean angiographical follow-up period was 9.3 months and the mean clinical follow-up period was 13.2 months. Patient’s characteristics are shown in Table 1. Nineteen out of 23 patients were male and mean age was 66.1 ± 11.4 years. Estimated glomerular filtration rate (GFR) was 70.8 ± 21.2 ml/min/1.73 m^2^. The diagnosis of patients showed 52.2% had stable effort angina, 43.5% silent myocardial ischemia and 4.3% acute coronary syndrome. Lesion location and lesion classification defined by ACC/AHA classification are shown in Table 2. Lesion location was 21.7% in left main trunk (LMT), 39.1% in the ostial left anterior descending artery (LAD), 34.8% in LAD proximal and 4.4% in right coronary artery (RCA). ACC/AHA classification of target lesion showed 4.3% of Type B1, 78.3% of Type B2, and 17.4% of Type C. Type A lesions were not included in this study. Procedural demographics are shown in Table 3. DCA–DCB procedures were successfully performed for all cases and no critical complications such as coronary perforation were reported. Mean procedural time was 123.6 ± 42.4 min and amount of contrast medium used was 207.5 ± 67.9 ml. The total number of cuts by DCA catheter was 30.5 ± 12.7 times and maximum inflation pressure was 4.5 ± 1.7 atm. Although there were cases of deep cutting and coronary dissection as minor complications (4.3 and 13.0%, respectively), no cases with minor complications received provisional stenting after DCA–DCB. QCA analysis and QCU analysis are shown in Tables 4 and 5. In QCA analysis before PCI, lesion length (LL) was 10.7 ± 3.8 mm, diameter stenosis (DS) was 61.6 ± 15.6%, reference vessel diameter (RVD) was 3.5 ± 0.6 mm, and minimal lumen diameter was 1.4 ± 0.6 mm. In QCU analysis before PCI, vessel CSA was 16.9 ± 4.9 mm^2^, luminal CSA was 3.6 ± 1.8 mm^2^, and plaque area was 77.3 ± 10.4%. Plaque area was reduced to 50.9 ± 9.2% immediately following DCA. In QCA analysis after DCB, DS was reduced to 16.3 ± 11.8% and acute gain was 1.8 ± 0.4 mm. Final plaque area after DCB was 47.3 ± 12.6%. Follow-up CAG revealed no cases with restenosis. QCA at follow-up CAG showed DS was 19.1 ± 11.0% and late loss was 0.16 ± 0.47 mm. QCU at follow-up CAG showed luminal CSA was nearly an equivalent level compared with those of at PCI (9.3 ± 3.3 mm^2^ at PCI and 9.5 ± 2.1 mm^2^ at follow-up CAG). In regard to APT after DCA–DCB, 5 of 23 patients were administered single APT using aspirin and dual-APT using aspirin and clopidogrel or prasugrel was given to 17 patients within an observational period. No patients caused MACE in this study.

**Table 1:** Baseline data of patients with DCA–DCB

Total	23
Male gender (*n*, %)	19 (82.6)
Age (years)	66.1 ± 11.4
Estimated GFR (ml)	70.8 ± 21.2
Clinical diagnosis	
Effort AP (*n*, %)	12 (52.2)
SMI (*n*, %)	10 (43.5)
ACS (*n*, %)	1 (4.3)
Coronary risk factors and comorbidities	
Hypertension (*n*, %)	17 (73.9)
Dyslipidemia (*n*, %)	17 (73.9)
Diabetes mellitus (*n*, %)	8 (34.7)
Smoking (*n*, %)	14 (60.8)
Prior MI (*n*, %)	3 (13.0)
Prior CABG (*n*, %)	0 (0)
Prior PCI (*n*, %)	10 (43.4)

*DCA* directional coronary atherectomy, *DCB* drug-coated balloon, *GFR* glomerular filtration rate, *AP* angina pectoris, *SMI* silent myocardial ischemia, *ACS* acute coronary syndrome, *MI* myocardial infarction, *CABG* coronary artery bypass grafting, *PCI* percutaneous coronary intervention

**Table 2:** Lesion location and lesion classification

Lesion locations	
LMT (*n*, %)	5 (21.7)
LAD ostial (*n*, %)	9 (39.1)
LAD proximal (*n*, %)	8 (34.8)
LCx ostial (*n*, %)	0 (0)
RCA (*n*, %)	1 (4.4)
Lesion classification (ACC/AHA classification)	
Type A (*n*, %)	0 (0)
Type B1 (*n*, %)	1 (4.3)
Type B2 (*n*, %)	18 (78.3)
Type C (*n*, %)	4 (17.4)

*LMT* left main trunk, *LAD* left anterior descending artery, *LCx* left circumflex artery, *RCA* right coronary artery

**Table 3:** Procedure demographics

DCA procedure demographics	
Success of DCA procedure (*n*, %)	23 (100)
Total number of cuts (times)	30.5 ± 12.7
Maximum cutting pressure (atm)	4.49 ± 1.74
Amount of plaque (mg)	24.3 ± 17.7
Radiation exposed dose (mSv)	1851 ± 1281
Procedure time (min)	123.6 ± 42.4
Amount of contrast medium (ml)	207.5 ± 67.9
Complication associated with DCA procedure	
Coronary perforation (*n*, %)	0 (0)
Coronary dissection (*n*, %)	3 (13.0)
Deep cutting (*n*, %)	1 (4.3)
DCB procedure demographics	
Balloon diameter (mm)	3.91 ± 0.28
Balloon length (mm)	21.3 ± 3.44
Balloon expandable pressure (atm)	7.78 ± 1.88

*DCA* directional coronary atherectomy, *DCB* drug-coated balloon

**Table 4:** QCA analysis

Pre-DCA	
Lesion length (mm)	10.7 ± 3.8
RVD (mm)	3.5 ± 0.6
MLD (mm)	1.4 ± 0.6
DS (%)	61.6 ± 15.6
Final	
RVD (mm)	3.8 ± 0.4
MLD (mm)	3.2 ± 0.6
DS (%)	16.3 ± 11.8
Acute gain (mm)	1.8 ± 0.4
Follow-up	
RVD (mm)	3.7 ± 0.3
MLD (mm)	3.0 ± 0.5
DS (%)	19.1 ± 11.0
Late loss (mm)	0.16 ± 0.47

*DCA* directional coronary atherectomy, *RVD* reference vessel diameter, *MLD* minimal lumen diameter, *DS* diameter stenosis

**Table 5:** QCU analysis

Pre-DCA	
Vessel CSA (mm^2^)	16.9 ± 4.9
Lumen CSA (mm^2^)	3.6 ± 1.8
Plaque area (%)	77.3 ± 10.4
Post-DCA	
Vessel CSA (mm^2^)	19.7 ± 4.5
Lumen CSA (mm^2^)	9.2 ± 1.3
Plaque area (%)	50.9 ± 9.2
Final	
Vessel CSA (mm^2^)	19.8 ± 4.3
Lumen CSA (mm^2^)	9.3 ± 3.3
Plaque area (%)	47.3 ± 12.6
Follow-up	
Vessel CSA (mm^2^)	20.3 ± 4.3
Lumen CSA (mm^2^)	9.5 ± 2.1
Plaque area (%)	52.0 ± 8.5

*DCA* directional coronary atherectomy, *CSA* cross-sectional area

In summary, none of the 23 patients treated with DCA–DCB in this study caused MACE including TVR and TLR during the follow-up periods.

## Discussions

This study demonstrated a favorable short-term outcome of PCI with IVUS-guided DCA followed by DCB, without any procedural-related critical complications, TLR, TVR and MACE during the follow-up periods.

To demonstrate the superiority of DCA to POBA, several clinical studies were conducted. The first clinical trial of DCA was CAVEAT trial conducted in 1991, in which the restenosis rate of DCA group at 6 months after PCI was not different statistically compared with those of POBA group [1]. This result seemed to be attributed to the incomplete and inadequate removal of plaque by angio-guided DCA, in which the plaque resection was limited and the selected device size was relatively small due to the concern of deep cutting of plaque which leads to critical events such as coronary perforation. Following the CAVEAT trial, the BOAT trial was also conducted to prove the superiority of DCA for POBA, in which an angiographically optimal DCA technique with large device to perform adequate cutting of plaque, post-dilatation with full size conventional balloon and achievement of residual stenosis < 20%, was performed in 1994. Residual stenosis of the DCA group was significantly lower compared with those of the POBA group (14.7 vs. 28.1%) and angiographic restenosis defined as late diameter stenosis ≥ 50% was also significantly lower than those of the POBA group (31.4 vs. 39.8%) in the BOAT trial [2]. Subsequently, the ABACAS trial was conducted in 1994 to compare the outcome between PCI with only DCA and with DCA followed by POBA [4]. In the ABACAS trial, all DCA procedures were performed under the aid of IVUS (IVUS-guided DCA), whereas IVUS was used in only 12.9% of DCA cases in the BOAT trial. IVUS-guided DCA resulted in the most aggressive removal of plaque and greater reduction of residual plaque area after DCA compared with previous studies. In the result of the ABACAS trial, whereas plaque area after PCI with adjunctive POBA after IVUS-guided DCA was smaller than those with IVUS-guided DCA alone, the restenosis rate and TLR rate at 6 months after PCI were not statistically different between the 2 groups, which indicated that adjunctive POBA after DCA might be unnecessary if adequate and effective removal of plaque was achieved by IVUS-guided DCA. In addition, the restenosis rate and TLR rate of the ABACAS trial was markedly lower compared with previous clinical studies, which indicated the efficacy of IVUS during DCA for achieving an aggressive plaque volume reduction.

In this study, we examined the efficacy of adjunctive DCB after IVUS-guided DCA. Although the post-interventional plaque area was 47.3 ± 12.6% in our study, which was relatively larger compared with those of PCI with adjunctive POBA after DCA in the ABACAS trial (42.5 ± 10.3%), no cases incurred restenosis and TVR at follow-up CAG. Furthermore, it was shown that the luminal CSA at the time of follow-up angiography remained unchanged or even had the tendency of enlargement compared with post-interventional luminal CSA in our study. We speculated that these favorable results could be contributed to the effect of concomitant use of the DCB. DCB, in which an anti-proliferative drug was coated on the surface of a semi-compliant balloon and drug infiltrates homogenously to the vessel tissue with mechanical expansion by balloon inflation, had recently emerged as a device to overcome the limitations of stents such as in-stent restenosis, stent thrombosis and bleeding complications associated with the necessity of administration of antiplatelet drugs. The first application of DCB was for in-stent restenosis, that the effectiveness of DCB was revealed by some randomized trials [12]. DCB use for de novo lesions was examined subsequently and the effectiveness of DCB for small vessel diseases was reported [13], and, therefore, other lesion subsets such as bifurcation lesions, diffuse lesions and lesions of acute myocardial infarction have been considered for DCB treatment. Also, some researchers reported luminal enlargement and positive vessel remodeling after DCB at follow-up period, resulted from the efficacy of the anti-proliferative drug and plaque regression [14, 15]. DCB is the device to release the drug into vessel walls and not to dilate the lesion, and thus adequate lesion preparation using semi-complaint or non-compliant balloon is needed [16]. Although, whether preparation of DCA before DCB is more effective against the lesion preparation by balloon has remained unclear, there are some studies in fields of peripheral interventions reporting that the removal of plaque by directional atherectomy was effective for proliferation of drugs of DCB to vessel walls and DCA–DCB might be an effective strategy for peripheral intervention [17–19]. We believe that DCA–DCB might be also useful option for coronary intervention. In regard to APT, dual-ATP may be unnecessary or enough in a short term compared with PCI with stents, because DCA–DCB strategy leaves no metal. Although only 5 cases were received single APT in this study, all cases with single APT caused no coronary events. Thus, it is considered that the combination therapy of DCA with DCB is possibly an effective strategy if DCA is performed optimally.

## Study limitations

This was a single-center, retrospective observational study with a very small study population. The follow-up period in this study was not sufficient to evaluate the long-term outcome of DCA–DCB. Investigation about APT after DCA–DCB should also be required because whether dual-ATP is necessary and how long dual-ATP should be administered remains unclear. Furthermore, lesion orientation was predominantly the LMT and ostium or proximal LAD. To demonstrate the efficacy and safety of a DCA–DCB strategy, a multicenter randomized prospective study with large population is required.

## Conclusions

The present study preliminarily demonstrated that DCA–DCB, which is the strategy of avoiding leaving metallic stents in the body, is a possibly useful option of PCI for non-tortuous and non-severe calcified lesions in proximal to middle segment with relatively large vessel diameter and short length.
